# Superoxide Dismutase as a Novel Macromolecular Nitric Oxide Carrier: Preparation and Characterization

**DOI:** 10.3390/ijms131113985

**Published:** 2012-10-29

**Authors:** Ssu-Han Chen, Shih-Jiuan Chiu, Teh-Min Hu

**Affiliations:** 1 School of Pharmacy, National Defense Medical Center, Taipei 11490, Taiwan; E-Mail: kido9300630@yahoo.com.tw; 2 School of Pharmacy, Taipei Medical University, Taipei 11031, Taiwan; E-Mail: shihjiuan@yahoo.com

**Keywords:** superoxide dismutase, nitric oxide, *S*-nitrosation, *S*-nitrosothiols, *S*-nitrosoglutathione

## Abstract

Nitric oxide (NO) is an important molecule that exerts multiple functions in biological systems. Because of the short-lived nature of NO, *S*-nitrosothiols (RSNOs) are believed to act as stable NO carriers. Recently, sulfhydryl (SH) containing macromolecules have been shown to be promising NO carriers. In the present study, we aimed to synthesize and characterize a potential NO carrier based on bovine Cu,Zn-superoxide dismutase (bSOD). To prepare *S*-nitrosated bSOD, the protein was incubated with *S*-nitrosoglutathione (GSNO) under varied experimental conditions. The results show that significant *S*-nitrosation of bSOD occurred only at high temperature (50 °C) for prolonged incubation time (>2 h). *S*-nitrosation efficiency increased with reaction time and reached a plateau at ~4 h. The maximum amount of NO loaded was determined to be about 0.6 mol SNO/mol protein (~30% loading efficiency). The enzymatic activity of bSOD, however, decreased with reaction time. Our data further indicate that NO functionality can only be measured in the presence of extremely high concentrations of Hg^2+^ or when the protein was denatured by guanidine. Moreover, mildly acidic pH was shown to favor *S*-nitrosation of bSOD. A model based on unfolding and refolding of bSOD during preparation was proposed to possibly explain our observation.

## 1. Introduction

Nitric oxide (NO) is a short-lived biomolecule with multifaceted physiologic and pathophysiologic activities. *In vivo* NO is enzymatically formed by various types of nitric oxide synthases (NOS) [1]. Once formed, this gaseous free-radical molecule exerts its action through direct and indirect pathways [2]. While, for example, the direct mechanism involves direct binding of NO to the heme iron of soluble guanylyl cyclase (sGC), thereby resulting in enzyme activation, the indirect pathway requires reactions of NO with multiple molecular targets (e.g., oxygen, superoxide), which transforms NO into various reactive (unstable) intermediates, *i.e.*, reactive nitrogen and oxygen species [2]. Thus, the metabolic half-life of NO is very short (within seconds) [3]. *S*-nitrosothiols (RSNOs), however, are more stable metabolites of NO, which are NO adducts of endogenous low- and high-molecular-weight thiols [4,5]. RSNOs can be formed either via reacting of thiols with N_2_O_3_, an NO-derived reactive intermediate, or through direct combination of NO and thiyl radicals. In addition, transnitrosation between RSNOs and thiols forms other RSNOs [4,5]. Stamler *et al.*[6] have shown that NO circulates in plasma mainly as *S*-nitroso (SNO) proteins; the majority of which is SNO albumin. It has therefore been suggested that the relatively stable RSNOs serve as reservoirs or carriers of NO with which the biological action of NO can be regulated in a sustained manner [6,7]. Thanks to advances in bioanalytical methods, various RSNOs have been detected and identified in biological systems [8,9] and *S*-nitrosoproteome has recently received much attention in the fields of proteomics and nitric oxide research [10–13].

The pioneer work of Stamler *et al.*[14] demonstrated that several sulfhydryl (SH)-containing proteins (bovine serum albumin, tissue-type plasminogen activator (t-PA), cathepsin B) can be nitrosated under synthetic and physiologic conditions. The study further showed that SNO proteins are more stable than NO and exert prolonged NO-like effects of vasodilatation and platelet inhibition. Later, poly-SNO-BSA (*i.e.*, NO added to polythiolated bovine serum albumin) was synthesized by the same research group (the Loscalzo group) for the purpose of evaluating this compound as an effective NO donor in an animal model of vascular injury [15]. Moreover, Ewing *et al.*[16] were able to synthesize poly-SNO-BSA with >2-fold increase in NO loading capacity. The concept of using protein-based RSNOs for therapeutic delivery of NO was thus emerging. Recent progress of albumin-based NO delivery systems includes: (1) pharmacokinetic characterization of SNO-BSA in mice [17]; (2) polyethylene glycol (PEG)-conjugated poly-SNO-BSA showing increased *in vivo* stability and prolonged release of NO [18]; (3) using human serum albumin (HSA) as the NO carrier and optimization of synthetic procedures [19–24]; and (4) a novel SNO HSA dimer being developed and evaluated as a potential anti-tumor drug [25].

**Figure 1:**
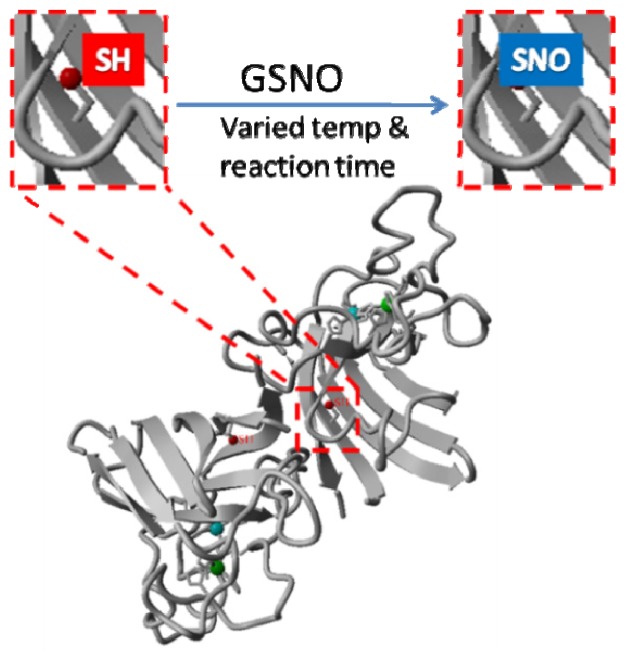
Schematic illustration of the preparation procedure for *S*-nitrosated bovine Cu,Zn superoxide dismutase (SNO-bSOD). The protein structure based on X-ray crystallographic data [27] for bovine Cu,Zn-SOD (PDB ID: 1sxb) was created with Yasara.

As reviewed above, macromolecules appear to be promising NO carriers that control the delivery of NO. In theory, virtually all proteins with free cysteine SH groups can be chemically modified into protein RSNOs. In practice, however, most efforts have been devoted to developing macromolecular NO delivery systems using the most abundant plasma protein—albumin (either of bovine or human origin). In the present study, we sought to synthesize and characterize a novel protein-based NO carrier using bovine Cu,Zn-superoxide dismutase (bSOD), which is a 32-kD homodimer antioxidant enzyme with superoxide-scavenging activity [26,27]. Each subunit of bSOD contains one free cysteine SH; therefore, there are two potential *S*-nitrosation sites in bSOD (Figure 1). Ideally, *S*-nitrosated bSOD (SNO-bSOD) should carry significant amount of NO (and control the delivery of NO), while still maintaining the dismutase activity. This dual-function feature of SNO-bSOD may have therapeutic implication in pathophysiologic conditions where overproduction of superoxide is coupled with low NO bioavailability, such as in ischemia-reperfusion injury [28–32].

## 2. Results and Discussion

### 2.1. Rationale and Synthetic Protocol for SNO-bSOD

As shown in Figure 1, bovine Cu,Zn-SOD (bSOD) is a homodimer containing one free cysteine SH (Cys6) in each subunit. Cys6 is located inside the β-barrel structure of bSOD, which is considered solvent inaccessible. Therefore, it is not apparent whether the SH moiety of Cys6 can be efficiently modified with an *S*-nitrosating agent. The first step of the present study, thus, was to determine reaction conditions suitable for preparation of *S*-nitrosated bSOD (SNO-bSOD). A simple synthetic procedure was employed in which bSOD (0.1 mM) was incubated with 5 mM *S*-nitrosoglutathione (GSNO) under varied experimental conditions (Figure 1). GSNO has been widely used as an *S*-nitrosating agent in preparing other SNO proteins [9,33–36], on the basis of *S*-transnitrosation occurring between GSNO and the SH functional groups of proteins.

### 2.2. Preparation, Separation and Basic Characterization of SNO-bSOD

**Figure 2:**
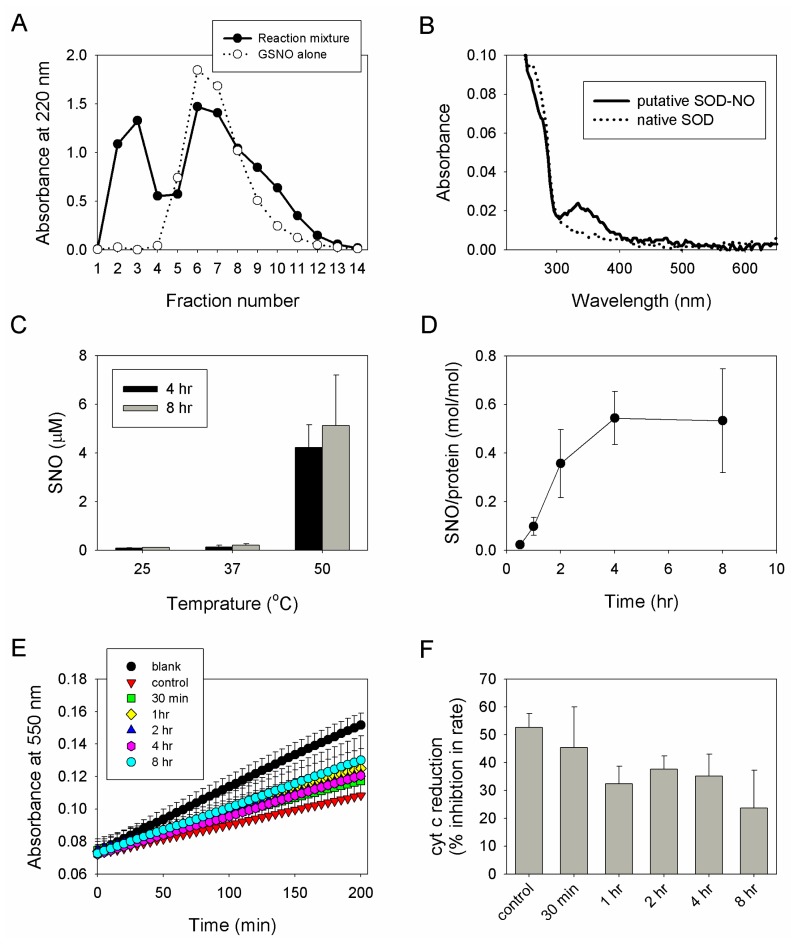
Separation and basic characterization of SNO-bSOD. (**A**) Elution profile of reaction mixture (bSOD + *S*-nitrosoglutathione (GSNO)) and GSNO alone (for comparison); (**B**) UV-vis spectra of SOD and SNO-bSOD; (**C**) Effect of reaction temperature on the extent of *S*-nitrosation (*n* = 3); (**D**) *S*-nitrosation efficiency (SNO mol/mol SOD) as a function of reaction time (at 50 °C; *n* = 3); (**E**) Kinetic of cytochrome c reduction in the absence or presence of native bSOD and of samples containing bSOD incubated with GSNO at 50 °C for various time periods; (**F**) Relative SOD activity expressed as percentage inhibition in the rate of cytochrome c reduction, as a function of incubation time (*n* = 3).

After incubating bSOD with GSNO, separation of macromolecules from excess unreacted GSNO was conducted by using gel filtration. According to the results shown in Figure 2A, fractions 2 and 3 (containing macromolecules) were then collected and pooled for further characterization. The UV-visible spectrum of the pooled sample was recorded, which shows a characteristic absorption peak at 334 nm, suggesting the presence of SNO group (Figure 2B). To determine the effect of varying synthetic conditions on *S*-nitrosation efficiency, SNO-bSOD was prepared at various temperatures and reaction times. The results indicate that significant *S*-nitrosation of bSOD occurred only at elevated temperature (50 °C, Figure 2C) for prolonged incubation time (>2 h, Figure 2D). *S*-nitrosation efficiency increased with reaction time and reached a plateau at ~4 h; at 50 °C, the maximum amount of NO loaded was about 0.6 mol SNO/mol protein (*i.e.*, ~30% loading efficiency) (Figure 2D). To determine the residual superoxide-scavenging activity of SNO-bSOD, inhibition of cytochrome c reduction by SNO-bSOD in a xanthine/xanthine oxidase system was measured. The results show that the enzymatic activity of SNO-bSOD decreased with reaction time (Figure 2E,F).

### 2.3. SNO-bSOD Is a Stable NO Carrier

**Figure 3:**
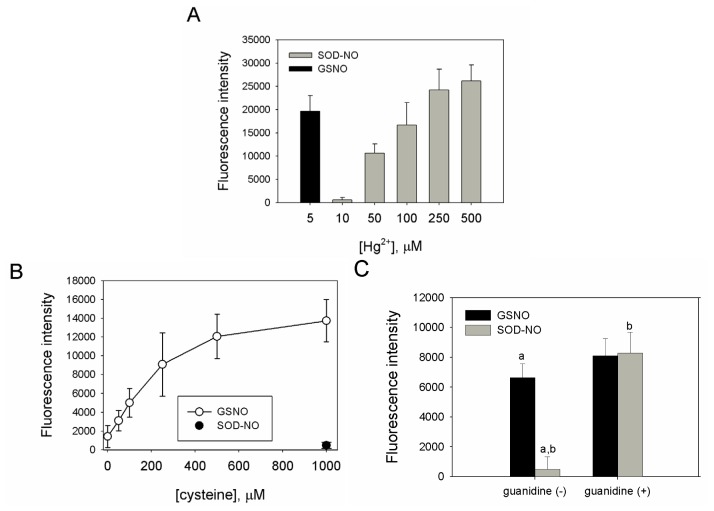
NO-release and reaction characteristics of SNO-bSOD. (**A**) Effect of mercuric ion (Hg^2+^) on NO release for SNO-bSOD and GSNO (*n* = 3). The sample containing SNO-bSOD (equivalent to 1.5 μM GSNO) was incubated in a PBS-based reaction buffer containing various concentrations of HgCl_2_. The mercury displaceable NO signal (fluorescence intensity) was detected using 2,3-diaminonaphthalene (DAN). The signal derived from 1.5 μM GSNO in the presence of 5 μM HgCl_2_ was included for comparison; (**B**) Effect of l-cysteine on NO release from SNO-bSOD and GSNO (*n* =3). GSNO was incubated in a reaction buffer containing various concentrations of l-cysteine and the derived NO signal (fluorescence intensity) was detected using DAN. For comparison, the NO signal derived from SNO-bSOD incubated with 1000 μM l-cysteine was included (solid symbol); (**C**) Effect of guanidine on cysteine-mediated NO release from SOD-NO and GSNO (*n* = 3). NO signal derived from GSNO or SNO-bSOD, which was incubated with L-cysteine in the absence or presence of guanidine chloride (3 M) for 3 h, was measured and compared. Statistical results: a. *p* < 0.001 for comparison between GSNO (without guanidine) and SNO-bSOD (without guanidine); b. *p* = 0.003 for comparison between guanidine-treated and untreated SNO-bSOD.

*S*-nitrosothiols (RSNOs) are comparatively more stable than other NO donors. Release of NO from RSNOs can be catalyzed by light, heavy metals and thiols [37–39]. In addition, thiols can facilitate decomposition of RSNOs via *S*-transnitrosation reaction [40–42]. To characterize the NO carrying and releasing capability of SNO-bSOD, the effect of mercuric ion (Hg^2+^) on the release of NO was investigated [43]. The results in Figure 3A show that, while low concentration (5 μM) of Hg^2+^ was sufficient to generate significant NO signals from GSNO, at least 50- to 100-fold Hg^2+^ was required to trigger comparable NO release from SNO-bSOD. This suggests that NO is stably incorporated into bSOD, thus rendering it insensitive to Hg^2+^ treatment.

Cysteine has been shown to mediate instability of RSNOs [44], resulting in generation of NO signals. To further demonstrate the stability of SNO-bSOD, cysteine-mediated production of NO signals from GSNO and SNO-bSOD was measured and compared. Figure 3B shows that, for GSNO, NO signals increased with increasing cysteine concentrations. In contrast, an insignificant NO signal was produced from SNO-bSOD, even at high [cysteine]. In the presence of guanidine hydrochloride (a protein-denaturing agent), however, cysteine can effectively mediate NO release from SNO-bSOD (Figure 3C), suggesting that in a denatured state of bSOD the SNO moiety becomes more accessible.

### 2.4. *S*-nitrosation Efficiency of bSOD Is Facilitated at Mildly Acidic pH

**Figure 4:**
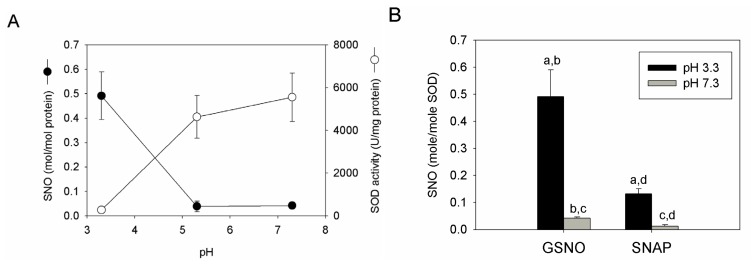
Effect of pH on *S*-nitrosation efficiency. (**A**) Effect of pH on GSNO mediated *S*-nitrosation of bSOD and residual SOD activity. Preparation of SNO-bSOD was conducted by incubating 5 mM GSNO with 0.1 mM bSOD at 50 °C for 4 h. The pH of reaction mixture was either left unadjusted (pH 3.3) or adjusted to 5.3 and 7.3. The final sample was assayed for SNO content and SOD activity, as described in the Experimental Section (*n* = 3); (**B**) Comparison including SNAP at pH 3.3 and 7.3 (*n* = 3). Statistical results: a. *p* = 0.025 for GSNO (pH 3.3) *vs.* SNAP (pH 3.3); b. *p* = 0.015 for GSNO (pH 3.3) *vs.* GSNO (pH 7.3); c. *p* = 0.002 for GSNO (pH 7.3) *vs.* SNAP (pH 7.3); d. *p* = 0.009 for SNAP (pH 3.3) *vs.* SNAP (pH 7.3).

At the concentration (*i.e.*, 5 mM) used for GSNO, the pH of the reaction mixture is 3.3. This is because GSNO is acidic in nature. Accordingly, the effect of pH on the *S*-nitrosation efficiency of bSOD was evaluated. The result shows that, if the pH of the reaction mixture was left unadjusted during preparation, a higher amount of SNO was obtained in bSOD; if, however, the pH was raised to around 5 and 7, the *S*-nitrosation efficiency was significantly reduced (Figure 4A). Similar pH effect was observed for preparations using *S*-nitroso-*N*-acetylpenicillamine (SNAP) as a nitrosating agent (Figure 4B). When the SOD activity was measured, it is of interest to note that low residual SOD activity was found for samples prepared under the low pH condition; at high pH values, however, the original SOD activity (*i.e.*, ~5000 U/mg protein) was almost preserved (Figure 4A).

### 2.5. Discussion

Previous studies have shown that incubation of proteins with an *S*-nitrosothiol (usually GSNO) led to the formation of *S*-nitrosated proteins [28–30]. It has also been shown that some proteins were more easily *S*-nitrosated than others [9,34]. The differential *S*-nitrosation reactivity may depend on the location of the free cysteine in proteins and the pK_a_ of the target SH group [10]; and conceivably, surface exposed cysteine is more labile to *S*-nitrosation than buried, solvent inaccessible ones. Since the target *S*-nitrosation site (Cys6) of bSOD is buried within the protein structure, the barrier for preparing SNO-bSOD can be immediately envisioned. Indeed, herein we report a preparation condition that is more stringent than those previously used for preparing *S*-nitrosated proteins using GSNO. Our study shows that an elevated temperature, a prolonged reaction time and an acidic medium were required for preparing *S*-nitrosated bSOD.

Reacting of bSOD with GSNO under the preparation condition (50 °C, pH 3.3) caused a significant loss of superoxide-scavenging activity of bSOD. Several factors may contribute to denaturation, thereby loss of enzymatic activity, of bSOD. Although bSOD is an enzyme with high thermostability [45,46], it is still possible that the preparation condition itself would cause denaturation of bSOD. To clarify this point, a sham experiment was conducted and the result shows that >90% of labeled SOD activity was recovered for native bSOD pretreated under the same preparation condition (but without GSNO) for 4 h. Thus, the SOD activity was not significantly affected by the incubation condition per se. However, low pH may cause a reversible, conformational change of bSOD. For example, Cu,Zn-SOD has been shown to undergo a structural change at pH 3 [47]. The structural transformation was reversible because the original structure restored after adjusting pH back to neutrality [47]. Accordingly, in the present study the protein structure of bSOD may be unfolded during incubation and refolded after separation by gel filtration (because of returning of pH to neutrality). The unfolding of bSOD may allow the buried cysteine to be exposed, which would then become *S*-nitrosated by reacting with GSNO. In addition, the exposed cysteine may further undergo GSNO-mediated oxidative modifications. Oxidation of cysteine may form dimerized proteins via intermolecular disulfide linkage, which would prevent the protein from refolding to its original state [46]. Moreover, in addition to *S*-nitrosation, GSNO can also elicit *S*-glutathiolation of proteins [33,34,48–52]; in the case of bSOD, *S*-glutathiolation of exposed Cys6 may impede protein refolding due to steric hindrance imposed by the addition of a glutathione moiety to Cys6. When the protein remained unfolded at neutral pH, its enzymatic activity would be reduced.

**Figure 5:**
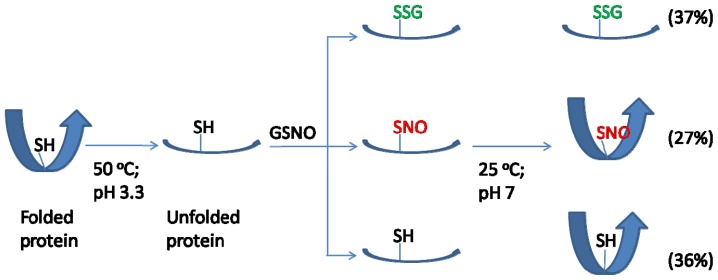
A proposed model for *S*-nitrosation of bSOD using GSNO as a nitrosating agent. The model assumes that bSOD unfolded at acidic pH and refolded at neutral pH. The numbers in parentheses represent the measured or estimated fractions of various possible bSOD species existing in the final reaction mixture. SH: unmodified Cys SH; SNO: *S*-nitrosated species; SSG: *S*-glutathiolated species.

One important feature for *S*-nitrosation of bSOD was that it occurred with higher efficiency at a high-temperature and low-pH condition (Figures 2D and 4), and even at such a condition the highest extent of *S*-nitrosation obtained was only about 20%–30% (*i.e.*, each mol bSOD contains 0.5–0.6 mol SNO). Obviously, *S*-nitrosation was even less efficient at lower reaction temperatures (25 or 37 °C) or at higher pH values (Figures 2C and 4). This is in contrast to what has been reported for other proteins. For example, using GSNO as a nitrosating agent, *S*-nitrosation with higher efficiency has been demonstrated for several proteins [33–35] under mild reaction conditions (e.g., room temperature and reaction time ≤1 h). Another feature of SNO-bSOD is that NO functionality can only be detected in the presence of extremely high concentrations Hg^2+^ (Figure 3A) or when the protein was denatured by guanidine (Figure 3C), suggesting that the SNO moiety is well protected within the protein structure. Therefore, based on the two features, a model that requires unfolding and refolding to occur during the preparation of SNO-bSOD was proposed (Figure 5), which would help understand the complex chemistry of SNO-bSOD.

First, the model assumes that it is difficult for GSNO to access, and thus to react with, the buried Cys6 of bSOD when the protein is in its native, folded state. Thus, the model proposes that bSOD should be in an unfolding state (*i.e.*, the rate determining step) such that Cys6 can be exposed and accessed by GSNO. This notion may be supported by the observation that a mildly acidic condition, at which bSOD has been shown to undergo structure transformation [47], was required to produce significant *S*-nitrosation. Considering the effect of pH on the stability and reactivity of RSNOs, the study of Liu *et al.*[53] showed that the stability of RSNOs was not dependent on pH. In addition, the rate of *S*-transnitrosation between an RSNO and a thiol increased with increasing pH [53]. Accordingly, if a limiting step such as unfolding of protein structure did not operate, one might expect a comparable or greater extent of *S*-nitrosation of bSOD occurring at higher pH (e.g., pH 7.3 *vs.* pH 3.3). Our result showing just the opposite is true suggests that *S*-nitrosation was enhanced by acid-mediated unfolding of bSOD.

Second, since maximally ~30% of the free cysteine in bSOD can be *S*-nitrosated, it is tempting to measure the amount of free SH remained in bSOD, which was done by a DTNB-based assay under a protein denaturing condition [45]. The result shows that only 36% of cysteine residues in SNO-bSOD were recovered as unmodified, free SH groups. Accordingly, about 1/3 of cysteine, which was neither *S*-nitrosated nor remaining unmodified, was unreactive to DTNB and was therefore considered as oxidized thiol species—possibly in an *S*-glutathiolated form [34] (Figure 5). The mechanism underlying the reaction of protein thiols with RSNOs is complicated and has not been fully understood. One of the possible pathways that lead to SNO formation is via *S*-transnitrosation (reaction 1 [42,53,54]) between GSNO and Cys6 of bSOD (possibly in its unfolded state).

(1) bSOD-SH+GSNO↔bSOD-SNO+GSH

An alternative pathway depends on NO release from GSNO, followed by reaction of the thiol group with NO-derived nitrosating species (e.g., N_2_O_3_). The latter mechanism, however, may play a minor role, because negligible SNO formation (~0.01 mol SNO/mol protein) was observed when bSOD was incubated with PAPA NONOate, a direct NO donor. Thus, under the premise that *S*-transnitrosation mainly operates in our system, low *S*-nitrosation efficiency may be attributed to low thiol reactivity towards GSNO at acidic pH, because thiolates were believed to be the major species participating in *S*-transnitrosation [41]. Alternatively, it is possible that bSOD was incompletely unfolded during incubation with GSNO, which may further hinder the *S*-nitrosation reaction. Furthermore, *S*-glutathiolation may occur via reaction 2 (direct glutathiolation) and reaction 3 (denitrosation of protein SNO) [34,48,55]; both reactions, while promoting the formation of *S*-glutathiolated species, can cause further reduction in *S*-nitrosation.

(2) bSOD-SH+GSNO→bSOD-SSG+HNO

(3) $$\text{bSOD}-\text{SNO}+\text{GSH}\to \text{bSOD}-\text{SSG}+{\text{NO}}^{-}$$

Finally, the model further proposes that refolding occurred for the SNO and unmodified bSOD species (in the final solution with neutral pH), but not for the *S*-glutathiolated species, based on the assumption that refolding is hindered by attaching a large molecule (*i.e.*, glutathione) to the sulfur atom. The refolding hypothesis may be supported by our finding that the NO functionality can only be revealed under a protein denaturing condition. Moreover, as discussed above, losing refolding capability of bSOD may partially account for the loss of its enzymatic activity.

The original idea of the present study was to prepare a dual-function SOD-based NO donor which can deliver NO and also act as a superoxide scavenger. Ideally, the proposed SOD-based NO donor could boost the cellular bioavailability of NO by simultaneously releasing NO and removing superoxide, given that NO rapidly react with superoxide to form a toxic metabolite, peroxynitrite. Accumulating evidence has shown that peroxynitrite is produced during ischemia and reperfusion and that reperfusion injury has been linked to peroxynitrite [30]. Remarkably, NO alone is protective against ischemia-reperfusion injury [30]. Thus, strategies to simultaneously increase NO and decrease superoxide levels in tissues may have significant therapeutic implications in ischemia-reperfusion injury. Although to engineer SOD into a bifunctional NO donor—which to the best of our knowledge is unprecedented—seems an attractive and feasible strategy, several factors or obstacles should be considered. First, the proposed NO donor should stably contain the NO molecule and slowly release it, because too quick a release of NO could result in the formation of peroxynitrite, in the milieu where superoxide is concomitantly produced. Second, the incorporation of NO may interfere with the superoxide scavenging activity of SOD. In this regard, the NO molecule should be attached to sites away from the catalytic center of SOD. Third, the cellular permeability of SOD-NO composite should also be considered. For Cu,Zn-SOD, suitable delivery systems may be needed to increase cellular membrane transport [56–59]. In the present study, we chose bovine Cu,Zn-SOD as the model SOD mainly because bovine Cu,Zn-SOD is a very stable enzyme and is more resistant to inactivation by nitrosative/oxidative stress, compared with other types of SOD (e.g., Mn-SOD) [60,61]. Cu,Zn-SOD of human origin may also be used, which is expected to result in higher NO loading efficiency because it contains an extra surface-exposed cysteine SH group per subunit. However, the ensuing surface-exposed SNO may be more reactive, thereby releasing NO in an unfavorable fashion.

Protein *S*-nitrosation mediated by abnormal NO signaling and excessive oxidative stress has been implicated in many pathophysiological conditions including neurodegenerative diseases [62–65]. For example, *S*-nitrosation of protein disulfide isomerase (PDI) has been shown to impair PDI chaperone/protein folding function, causing protein misfolding and ER stress, which may contribute to neuronal cell injury/death in Alzheimer’s disease (AD) [66]. Currently, it is yet to determine whether Cu,Zn-SOD can be *S*-nitrosated *in vivo*. Under extreme nitrosative stress, the surface-exposed Cys111 of human Cu,Zn-SOD [67,68] may be a target of *S*-nitrosation, which remains to be identified. In contrast, *S*-nitrosation of the buried cysteine in bovine or human Cu,Zn-SOD may be less likely to occur, especially when proteins are in the native, folded state. The result of our *in vitro* study, however, suggests that *S*-nitrosation of buried cysteine is attainable in the unfolded state of proteins. *In vivo*, unfolded mutant Cu,Zn-SOD has been associated with amyotrophic lateral sclerosis (ALS), a fatal neurodegenerative disease [69–71]. A testable hypothesis related to ALS therefore is that *S*-nitrosation of unfolded Cu,Zn-SOD may facilitate protein oxidation, leading to the formation of toxic protein aggregates.

## 3. Experimental Section

### 3.1. Chemicals

Bovine erythrocyte Cu,Zn-superoxide dismutase (bSOD), *S*-nitrosoglutathione (GSNO), *S*-nitroso-*N*-acetylpenicillamine (SNAP), 2,3-diaminonaphthalene (DAN), cytochrome c, xanthine, xanthine oxidase, mercury (II) chloride, copper (II) chloride, ferrous sulfate, diethylenetriamine pentaacetic acid (DTPA), l-cysteine, guanidine HCl, and 5,5′-dithiobis-(2-nitrobenzoic acid) (DTNB) were purchased from Sigma-Aldrich (St. Louis, MO, USA). The Pierce 660 nm Protein Assay Reagent was purchased from Thermo Scientific (Rockford, IL, USA).

### 3.2. Synthesis of SNO-bSOD

*S*-nitrosated bSOD was prepared using GSNO as a nitrosating agent with a molar ratio (bSOD: GSNO) of 1:50. Initially, to determine better reaction conditions, bSOD (0.1 mM) was incubated with 5 mM GSNO (in aqueous solution) at 25, 37 or 50 °C for various time periods (0.5–8 h). The pH of the reaction mixture was measured to be 3.3. All reactions were protected from light. After incubation, separation of excess unreacted GSNO from protein was performed by passing the reaction mixture through a Sephadex G-25 gel filtration column (1.6 × 2.5 cm; GE Healthcare Life Sciences), and the eluent used was 0.01 M phosphate buffered saline (PBS) containing 0.5 mM DTPA. Fraction collection was conducted, followed by spectrophotometric determination at 220 nm. The first few fractions with high UV absorbance were pooled and stored at −20 °C until further analysis. To investigate the effect of pH on *S*-nitrosation efficiency, the pH of incubation solution was either left unadjusted (pH 3.3) or adjusted to 5.3 and 7.3. In addition, synthesis was also conducted using SNAP and PAPA NONOate.

### 3.3. Spectroscopic Characterization of SNO-bSOD

The SNO-bSOD sample was concentrated using ultrafiltration (Amicon ultra-0.5, Millipore, Billerica, MA, USA). The UV-vis absorption spectra were recorded for native bSOD and SNO-bSOD (both at 120 μM), respectively, using the NanoDrop ND-1000 spectrophotometer (Thermo Scientific, Wilmington, DE, USA).

### 3.4. Fluorometric Determination of *S*-nitrosation Efficiency

The assay of SNO was performed according to a previous report [43] with slight modification. Briefly, the SNO-bSOD sample was divided into two equal aliquots. To one sample, an aliquot of acidic 2,3-diaminonaphthalene (DAN) solution (prepared in 0.62 N HCL) was added to a final concentration of 30 μM; to the other, in addition to DAN, HgCl_2_ (final conc. = 50 μM) was also added. The samples were then incubated at room temperature in the dark for 30 min. After incubation, an aliquot of 8.5 N NaOH was added to each sample to terminate the reaction, and the fluorescence intensity was subsequently measured with a fluorescence microplate reader (Infinite M200, Tecan Austria GmbH), with an excitation wavelength of 363 nm and an emission wavelength of 450 nm. The quantity of SNO was determined by subtracting the fluorescence intensity generated in the absence of HgCl_2_ (background) from that generated in the presence of HgCl_2_. A standard curve was constructed using GSNO as the standard.

### 3.5. Determination of Protein Concentration

The amount of SOD protein was determined by a dye-metal-based colorimetric protein assay [72]. The assay was conducted in a microplate format and a commercially available reagent (Pierce 660 nm Protein Assay Reagent) was used. After mixing the sample with the reagent at room temperature for 5 min, absorbance at 660 nm was measured using a microplate reader (PowerWave XS, Bio-Tek, Winooski, VT, USA). A standard curve was constructed using native bSOD.

### 3.6. Measurement of SOD Activity

SOD activity was assayed spectrophotometrically (Shimadzu UV-2450, Kyoto, Japan) by the xanthine/xanthine oxidase/cytochrome c method [26]. The reaction mixture contained 10 μM cytochrome c and 30 μM xanthine in 50 mM potassium phosphate buffer containing 0.1 mM EDTA, at pH 7.8 and 25 °C. Reactions were started by adding xanthine oxidase (1.2 milliunits) to the reaction mixture, which caused an increase in absorbance at 550 nm at a rate of about 0.025 absorbance unit/min (*i.e.*, blank rate). The assay condition allowed one unit of native bSOD to cause a 50% inhibition in the reduction rate of cytochrome c (*i.e.*, a control rate of about 0.0125 absorbance unit/min). To determine the residual SOD activity in samples, an aliquot of the sample (equivalent to 1 unit of SOD) was added to the reaction mixture, followed by adding xanthine oxidase to start the reaction, and then the rate of cytochrome c reduction was measured. The activity was expressed either as percentage inhibition in the reduction rate or as specific activity (unit/mg protein).

### 3.7. Characterization of NO Release at Neutral pH

To characterize Hg^2+^-induced release of NO functionality (NO*_x_*), diluted SNO-bSOD samples (equivalent to 1.5 μM SNO) were incubated with various concentrations (up to 500 μM) of HgCl_2_ in PBS (0.01 M, pH 7.4) at 37 °C in the dark for 30 min. The incubation time was determined according to preliminary kinetic experiments showing that Hg^2+^ triggered almost complete NO release within 30 min. At the end of incubation, an aliquot of acidic DAN solution was added (final DAN conc. = 30 μM) and after 10 s the reaction was terminated by the addition of 10 μL 8.5 N NaOH. The fluorescence intensity was measured using a fluorescence microplate reader (excitation: 363 nm; emission: 450 nm).

To characterize cysteine-induced release of NO*_x_*, GSNO (1.5 μM) or diluted SNO-bSOD samples (equivalent to 1.5 μM SNO) were incubated with l-cysteine (up to 1000 μM) in PBS (0.01 M, pH 7.4, containing 0.5 mM EDTA) at 37 °C in the dark for 60 min. At the end of incubation, an aliquot of acidic DAN solution was added (final DAN conc. = 30 μM) and after 10 s the reaction was terminated by the addition of 10 μL 8.5 N NaOH. The fluorescence intensity was measured as indicated above. The effect of guanidine on cysteine-mediated NO*_x_* release was investigated by adding guanidine hydrochloride (3 M) in the reaction solution (containing 1 mM l-cysteine), followed by incubation for 3 h and fluorometric determination.

### 3.8. Quantification of Free Cysteine

The determination of free cysteine in SNO-bSOD was conducted under a denaturing condition [45,73]. Briefly, SNO-bSOD was reacted with DTNB (5,5′-dithiobis-(2-nitrobenzioc acid)) in a reaction buffer (pH 7.4) containing guanidine hydrochloride at room temperature. The final reaction mixture contained: 5 μM SOD, 100 mM potassium phosphate, 2 mM EDTA, 0.5 mM DTNB, and 6 M guanidine hydrochloride. After initiating the reaction by addition of DTNB, the absorbance at 412 nm was measured at 10-min intervals until no significance increase in absorbance was observed (~3 h). Standard curves were constructed using l-cysteine. Under the same experimental condition, the amount of free cysteine residues in bSOD was determined to be about 0.84 residues per subunit, which is consistent with previous literature reports [45,73].

### 3.9. Data Analysis

Data are expressed as mean ± standard deviation from three replicates. The Student’s *t*-test was used to evaluate statistical significance; results were considered significant when *p* < 0.05.

## 4. Conclusions

In the present study, we have for the first time prepared and characterized *S*-nitrosated bovine Cu,Zn-SOD (SNO-bSOD). Our result demonstrates that a stringent condition (50 °C and pH 3.3) was required to render bSOD partially *S*-nitrosated. In addition, we show that SNO-bSOD was a stable NO carrier and the NO functionality can only be detected under a denaturing condition. Finally, we propose an unfolding-refolding hypothesis to possibly explain our observation.
